# Benchmarking Multi-Rate Codon Models

**DOI:** 10.1371/journal.pone.0011587

**Published:** 2010-07-21

**Authors:** Wayne Delport, Konrad Scheffler, Mike B. Gravenor, Spencer V. Muse, Sergei Kosakovsky Pond

**Affiliations:** 1 Department of Pathology, University of California San Diego, San Diego, California, United States of America; 2 Department of Mathematical Sciences, University of Stellenbosch, Stellenbosch, South Africa; 3 School of Medicine, University of Swansea, Swansea, Wales; 4 Department of Statistics, North Carolina State University, Raleigh, North Carolina, United States of America; 5 Department of Medicine, University of California San Diego, San Diego, California, United States of America; BC Centre for Excellence in HIV/AIDS, Canada

## Abstract

The single rate codon model of non-synonymous substitution is ubiquitous in phylogenetic modeling. Indeed, the use of a non-synonymous to synonymous substitution rate ratio parameter has facilitated the interpretation of selection pressure on genomes. Although the single rate model has achieved wide acceptance, we argue that the assumption of a single rate of non-synonymous substitution is biologically unreasonable, given observed differences in substitution rates evident from empirical amino acid models. Some have attempted to incorporate amino acid substitution biases into models of codon evolution and have shown improved model performance versus the single rate model. Here, we show that the single rate model of non-synonymous substitution is easily outperformed by a model with multiple non-synonymous rate classes, yet in which amino acid substitution pairs are assigned randomly to these classes. We argue that, since the single rate model is so easy to improve upon, new codon models should not be validated entirely on the basis of improved model fit over this model. Rather, we should strive to both improve on the single rate model and to approximate the general time-reversible model of codon substitution, with as few parameters as possible, so as to reduce model over-fitting. We hint at how this can be achieved with a Genetic Algorithm approach in which rate classes are assigned on the basis of sequence information content.

## Introduction

The inference of selection within protein coding genes has benefited greatly from both the development of a probabilistic framework for phylogenetics [1] and codon models (see [2], [3] for recent reviews). Indeed, the use of codon models has facilitated the identification of selection occurring at sites [4], [5] and along lineages [6], [7]. A fundamental feature of all codon models is that they assign different rates to synonymous (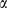) and non-synonymous (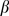) substitutions. Each rate is shared within the class, hence 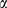 is the “average” of synonymous substitution rates for all possible one-nucleotide substitutions that don't change the amino acid, and 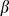 is its non-synonymous analog. This parameterization permits inference of selection at sites/lineages where non-synonymous substitutions occur at higher rates than do synonymous substitutions (*i.e.* 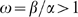), but is nonetheless biologically implausible. The nearly universal modeling assumption that all non-synonymous substitutions occur at the same rate is contrary to evidence that residue exchangeabilities are dependent on the physicochemical properties of amino acids (e.g. [8]). Indeed, protein models derived by estimating the relative rates of amino-acid substitution in large protein databases consistently show dramatic differences in the relative replacement rates of different residues (e.g. [9]–[11]).

To improve the biological realism of codon models, several recent studies proposed substitution models in which non-synonymous substitution rates depend on the residues (multi-rate codon models). These models divide non-synonymous substitution pairs into multiple categories, and infer substitution rates assuming they are shared by all the pairs in the same category. Current multi-rate models include (i) a generic empirical codon model (ECM) estimated by maximum likelihood from the alignments of 7,332 protein families [12], (ii) a linear combination of amino acid properties model (LCAP) that expresses the exchangeabilities of codons as a function of the physicochemical distances between the the amino acids which they encode [13], (iii) a model in which amino acid substitution biases are incorporated into codon models by weighted partitioning of empirically-derived amino acid substitution rates [14], (iv) the assignment of amino acids to physico-chemical property classes and the estimation of substitution rates within and between these classes [15], [16] and (v) a Bayesian approach (for models of protein evolution) which assigns substitutions to classes with a Dirichlet process [17]. The purpose of these models is to incorporate biologically realistic substitution processes into codon models that are frequently used for the estimation of selective pressure. If amino acids are subdivided into classes based on a physico-chemical property (as in [15], [16]), selection for property (such as polarity) preservation may be measured as the decreased rate of non-synonymous substitution between versus within classes. Alternatively, if amino acid pairwise substitutions are subdivided into classes, we can determine whether there is preferential replacement of a subset of amino acids, suggesting directional selection. Most frequently, a newly proposed multi-rate codon model is compared to the single-rate model and a statistically significant improvement in fit is obtained to demonstrate its utility. However, the approaches have not been rigorously compared against each other and it is unclear how highly each of the models rank in the space of all possible substitution models. Our current work is focussed on inferring multi-rate models of codon evolution from alignments, and in particular the development of a Genetic Algorithm [18] for multi-rate codon model selection. In so doing we have asked the question “what is the appropriate reference model to which new models should be compared”? In this note we demonstrate that the single rate (SR) model is an inappropriate baseline model, and rather advocate the use of a codon general time-reversible (REV) model. This changes the focus of model comparison from how much better a new model is than a weak model (SR), to how well a new model approximates the most general model (REV).

## Materials and Methods

We consider the class of time-reversible codon substitution models which allow a single nucleotide to be substituted instantaneously, with SR being the simplest and REV being the most general, and models such as LCAP occupying an intermediate range. The rate matrix 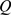 for such a model consists of elements 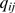 that encode the rate with which sense codon 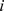 is replaced with sense codon 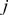:
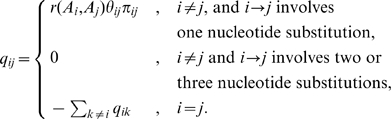
The three multipliers represent (i) the exchangeability of the amino-acid residues encoded by codons 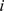 and 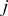, 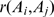 (note that because the model is time reversible 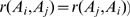), (ii) nucleotide mutational biases, 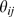, and (iii) equilibrium frequency parameters, 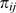, estimated by 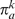 – the frequency of the target nucleotide 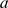, assuming the substitution replaces the 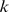-th nucleotide, *i.e.* using the frequency parameterization of [19]. All model parameters are estimated by maximum likelihood. For all models, all synonymous rates (where 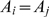) are set to 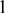. The SR model assigns a single parameter 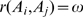 to all substitution rates. In this case 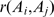 is the same as the commonly estimated 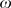 or 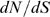 selection parameter. The REV model is obtained by allowing each pair of distinct amino-acid residues to have an independent rate (*i.e* 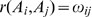). Assuming the universal genetic code, 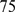 out of 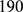 possible residue pairs can be exchanged via a single nucleotide substitution, hence this model will have 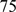 estimable non-synonymous rate parameters.

Models of intermediate complexity, which we hypothesize will be supported by biological data, are obtained when the number 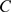 of non-synonymous rate parameters is between 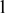 and 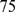, *i.e.* some residue pairs are exchanged at the same rates, but there may be several of these non-synonymous rates. We define the number of rate classes *a priori* (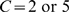) and assign substitutions to rate classes randomly with uniform probability, *i.e.* on average the same number of rates are allocated to each class. Note that we do not randomly assign the amino acids themselves to rate classes, but rather each of the 75 pairwise substitutions to rate classes (i.e 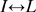, 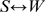, etc.) Previous approaches [15], [16] have assigned amino acids to classes on the basis of physicochemical properties and estimated substitution rates within- and between these amino acid classes. These models, however, are limited by their enforced transitivity of rates (i.e. the requirement that if 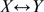, and 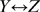 are in the same rate class, then so is 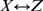). Because the genetic code itself is not transitive, i.e. one can easily find triplets of amino-acid residues (for instance 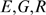), where 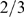 pairs can be exchanged with a single nucleotide substitution, but the last pair requires two. Enforcing the same substitution rates between one- and two-step nucleotide substitutions is not easily justified. Theoretically, multi-rate codon model selection could be based on the random assignment of amino acid substitutions to rate classes, however, this approach is infeasible given that there are 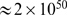 models with 5 rate classes. Rather we simply generate these random models for the purpose of demonstrating how easily the single rate model is improved upon. An alternative is to assign pair-wise amino acid substitution rates to classes using a data-driven approach. Here, we include results for such an approach based on a Genetic Algorithm (GA), which we describe in a separate manuscript [20]. We compare the fit of random models to the SR, ECM, LCAP, GA and REV models using 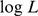 scores and likelihood ratio tests (when appropriate). For the comparison of random versus SR models we generated 100 instances of the random model with 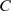 classes. Because the SR model is nested within any random model, a likelihood ratio test with 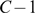 degrees of freedom can be used to assess significance.

We chose three representative empirical data sets for our model fit comparisons, namely (i) the PF00803 Pandit [21] alignment (3A/RNA2 movement protein family, 13 sequences, 277 codons), a rhodopsin dim-vision protein alignment (38 sequences, 330 codons) from [22], and an HIV-1 group M partial polymerase gene alignment (98 non-recombinant sequences, 541 codons).

## Results and Discussion

For all three alignments, the SR model could be rejected in favor of a random multi-rate model in the majority of cases with the likelihood ratio test at 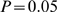 level (Table 1). For models with two rate classes, significantly improved model fit was evident in at least 43 and up to 80 of the 100 random models (15 and 66 with Bonferroni correction). Models with 5 rate classes showed significantly improved model fit for 96 to 100 of the 100 permutations. Our analysis demonstrates that given a sufficiently large alignment, effectively *any random* multi-rate model with 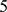 rate classes is preferred to the SR model. This observation raises serious doubts as to the utility of a single rate model as a benchmark for model comparison.

**Table 1:** Comparison of single rate versus random models for 3 alignments.

alignment	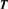	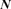	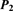	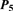
Pandit PF00803	13	27	43 (15)	96 (80)
Rhodopsin	38	330	80 (66)	100 (99)
HIV-1 group M *pol*	98	541	80 (56)	99 (96)

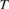
 = number of taxa, 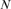 = number of sites, 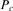 = number of random permutations out of 100 which showed significantly improved fit over the 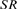 model (Likelihood Ratio Test, 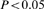). Numbers in parentheses are based on Bonferroni corrected 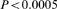.

As an analogy, consider the family of nucleotide models, where JC69 [23] and the general-time reversible (GTR) model [24] are representative of the two extremes of model space, where model space is defined by the number of pair-wise nucleotide substitution rates. Whilst one cannot argue against the GTR model being the most representative of the mutation process, it is seldom selected as the best fitting model. Indeed, of approximately 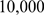 sequence alignments submitted to Datamonkey [25] for nucleotide model selection, not a single one supported the GTR model over other models. Note that the model selection procedure in Datamonkey [26] examines all 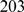 nucleotide time-reversible models. This approach is clearly infeasible for codon models, since there are 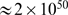 codon models with 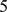 rate classes, for example. The most frequently selected model (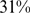 of cases) was the two parameter HKY85 model [27]. This does not suggest that HKY85 is the most biologically plausible, but rather the best approximation to the GTR given limited sample size.

**Figure 1:**
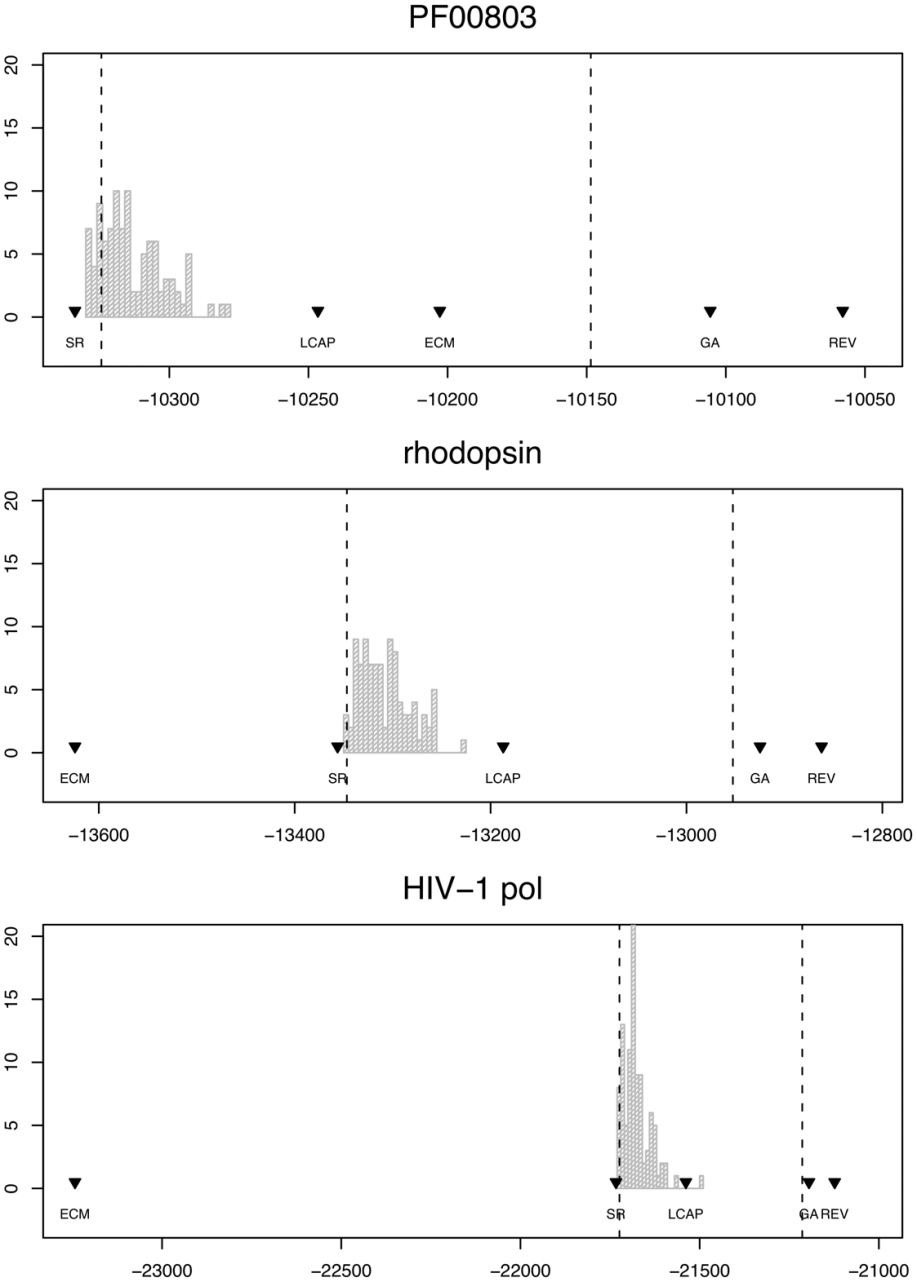
Distribution of log likelihood scores for 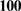 multi-rate models where amino-acid substitutions are assigned randomly to 5 non-synonymous classes. The fit of single rate (SR), linear combination of amino acid properties (LCAP), empirical codon model (ECM), Genetic Algorithm (GA) and the general reversible model (REV) are shown as upside-down triangles. Number of rate classes inferred in the GA are 3, 4 and 5 for PF00803, rhodopsin and HIV-1 *pol*, respectively. Dashed lines indicate the log likelihood required to (i) reject the single rate model in favor of a 5 rate model (left), and (ii) reject a 5 rate model in favor of REV (right). All models were fitted using maximum likelihood estimates of position-specific nucleotide frequencies.

Consequently, we should assess codon models not by whether or not they outperform the single rate model, but rather by how they measure up against the general codon model (*i.e.* REV). We demonstrate using log likelihood scores. As previously shown [12], [13], both LCAP and ECM models fit better than a single rate model, at least for the Pandit alignment (Figure 1). Since SR is nested within LCAP, the improvement in log likelihood score follows by necessity. However, a glance at Figure 1 should convince the reader just how trivially easy it is to outperform the SR model. Comparison of models using BIC (Table 2) indicates the GA to be the best model for all three alignments. As evident in comparison of log likelihoods (Figure 1), BIC scores for random models also indicate improved fit over the single rate model. ECM is ranked second when fitted to one of the alignments used in ECM estimation (PF00803), yet fits rhodopsin and HIV-1 alignments *worse* than the single rate model, suggesting it may be impossible to derive a generalist empirical codon model.

**Table 2:** Comparison of empirical model fits using BIC.

# rate parameters	SR	ECM	LCAP	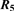	GA	REV
	1	0	5	5	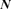	75
Pandit PF00803	20978.6	20667.2	20844.9	20970.3	**20538.3 (3)**	21032
Rhodopsin	27514.5	27994	27223.6	27454.6	**26680.2 (4)**	27224.6
HIV-1 *pol* Group M	45729.2	48683.2	45394	45658.5	**44696.1 (5)**	45314.1

The best model (with smallest BIC) is shown in boldface. The BIC for 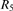, the model in which amino acid substitution pairs are randomly assigned to one of 5 rate classes, is estimated as the mean BIC over 100 permutations of rate class assignment. 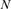 is the number of rate classes identified using a Genetic Algorithm model fitting procedure described in [20] and shown in parenthesis for each alignment. All models were fitted using maximum likelihood estimates of position-specific nucleotide frequencies.

When developing multi-rate models of codon evolution we should strive to not only beat the single-rate model, but also to approximate the REV model with the fewest possible parameters. Consider a multi-rate model with 5 independent rate parameters, as in LCAP. In this case we can plot the log-likelihood limits at which we reject a single-rate model (left hand dashed line in Figure 1), and at which we reject a 5-rate model in favor of the reversible model (right hand dashed line), say at 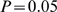 using the likelihood ratio test (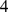 degrees of freedom in the first case, 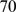 in the second). The performance of most multi-rate codon models thus far falls between these limits, *i.e.* the models improve upon the case of the single rate but can be rejected in favor of REV. We should construct multi-rate codon models that match the performance of REV in a statistical sense, with comparable likelihood scores, but with sufficiently few parameters to be computationally tractable and estimable from reasonable alignments. Only one model, the GA [20], achieves this in all cases. This model is set up so as to prevent over-parameterization, which is achieved by incrementing the number of non-synonymous rate classes, 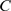, evaluating the fitness of a population of 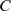-rate models using an appropriately chosen information criterion, and repeating until fitness is no longer improved with an increase in the number of rate classes. The fact that none of the 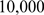 model selection analyses run on Datamonkey contained enough data to reject all simpler models in favor of a six parameter nucleotide GTR suggest that we should similarly focus our efforts in the codon space on models with a small number of rate classes, and investigate the space of candidate models thoroughly.

In conclusion, we have shown the single rate model to be a poor benchmark for model comparison, given that random models nearly always offer improved fit. We argue that the conceptual approach to codon model selection should instead focus on finding multi-rate models with a few parameters that can match the performance of REV, *i.e.* cannot be rejected in favor of REV, on alignments of biologically realistic size. Furthermore, our examples highlight the poor fit of “generic” empirical multi-rate models and suggest that new multi-rate models should be alignment specific. Whilst it is not advisable to fit a parameter rich REV model in practice, due to computational constraints and uncertainty in parameter estimates on small alignments, we should aim to derive the best model, given the limitations posed by the size of the alignment.
